# Evaluation of Volitional Swimming Behavior of *Schizothorax prenanti* Using an Open-Channel Flume with Spatially Heterogeneous Turbulent Flow

**DOI:** 10.3390/ani12060752

**Published:** 2022-03-17

**Authors:** Minne Li, Ruidong An, Min Chen, Jia Li

**Affiliations:** State Key Laboratory of Hydraulics and Mountain River Engineering, College of Water Resource & Hydropower, Sichuan University, Chengdu 610065, China; sculimn@stu.scu.edu.cn (M.L.); anruidong@scu.edu.cn (R.A.)

**Keywords:** potamodromous fish, fish behavior, hydropower, eco-hydraulics, fishway

## Abstract

**Simple Summary:**

A lack of information on fish volitional swimming behavior in response to various instream flow conditions may inhibit the development of an effective fishway. In this study, *Schizothorax prenanti,* Tchang, 1930 (*S. prenanti*), an endemic species in the Jinsha river basin, was tested for volitional swimming behavior in a self-designed open-channel flume. Quantified indices, describing preferred hydraulic characteristics, swimming strategies, and swimming speeds, were collected to analyze fish behavioral responses to turbulent flow. The results showed that *S. prenanti* preferred to select regions with low flow velocities (0.25–0.50 m/s) and turbulent kinetic energy (<0.05 m^2^/s^2^) and employed steady swimming behavior to search for flow velocities lower than the average current to conserve energy in low- and moderate-flow regimes. Additionally, the average and maximum burst speeds of *S. prenanti* were 2.63 ± 0.37 and 3.49 m/s, respectively. The aim of this study was to apply fish volitional swimming behavior data on the the design and optimization of fishways for specific target species, as well as a novel test method for other migratory fish species.

**Abstract:**

Effective fishway design requires knowledge of fish swimming behavior in streams and channels. Appropriate tests with near-natural flow conditions are required to assess the interaction between fish behavior and turbulent flows. In this study, the volitional swimming behavior of *S. prenanti* was tested and quantified in an open-channel flume with three (low, moderate, and high) flow regimes. The results showed that, when confronted with alternative flow regimes, *S. prenanti* preferred to select regions with low flow velocities (0.25–0.50 m/s) and turbulent kinetic energy (<0.05 m^2^/s^2^) for swimming, while avoiding high-turbulence areas. Moreover, *S. prenanti* primarily employed steady swimming behavior to search for flow velocities lower than the average current to conserve energy in low- and moderate-flow regimes. It is hypothesized that in regions with higher flow velocities, fish may change their swimming strategy from energy conservation to time conservation. Additionally, the average and maximum burst speeds of *S. prenanti* were 2.63 ± 0.37 and 3.49 m/s, respectively, which were 2.21- and 2.28-fold higher than the average (1.19 m/s) and maximum (1.53 m/s) burst speeds estimated from the enclosed swim chamber for fish of similar length. This study contributes a novel research approach that provides more reliable information about fish volitional swimming behavior in natural habitats, as well as recommendations for hydraulic criteria for fishways and the identification of barriers to fish migrations.

## 1. Introduction

Hydropower and other water abstraction facilities are primarily developed for renewable energy, irrigation, and flood control [1,2]. However, the habitat fragmentation of natural rivers, an ecological impact of hydropower projects, is considered to be a major threat to aquatic ecosystems worldwide, including those of migratory fish [3]. Often, these hydropower facilities create physical or flow barriers that may exceed the behavioral or physiological capability of specific or all species to pass [4]. The effects of fragmentation from hydraulic projects on migratory fish include migration route blocking, habitat disconnection, fish species isolation, turbine-related mortality, and potentially the extinction of some aquatic species [5]. Mitigation efforts such as the construction of fishways have been recognized as an effective management application for balancing water resource development and aquatic ecosystem conservation [6]. The design of efficient fishways has been inhibited by a poor understanding of the swimming behavior of migratory fish (e.g., route selection, behavior strategy, and swimming ability), as well as their responses to various hydrodynamic characteristics, such as flow velocity, vorticity, TKE, and Reynolds shear stress (RSS), in aquatic environments [7,8,9,10].

The spatial and temporal migratory movement of fish in natural rivers is strongly mediated by hydrodynamic environments, as well as other inherent and external factors [11]. Fish use fine-scale lower flow velocity and turbulence as navigation cues for downstream migration in rivers [8], since strong turbulence can interfere with their swimming behavior, resulting in reduced navigation stability and increased energy costs, stress, and injury risk [12,13]. Furthermore, when confronted with a variety of locomotory challenges, fish behavior results from a complex tradeoff ultimately linked to energy conservation and the benefits of various swimming strategies [14]. A previous study found that salmon preferentially migrated in nearshore and bottom regions with current speeds lower than the average speed of the river [15]. In higher flow velocity regions, rather than searching for lower-speed pathways, fish tend to exhibit unsteady swimming behavior to increase their swimming speed for time conservation [16].

Studies on different migratory fish species have revealed that turbulent flow affects not only path selection and swimming strategy, but also swimming speed [4,17]. The traditional method for assessing the swimming ability of fish, developed by Brett (1964) [18], evaluates the movement of fish confined in an enclosed flow chamber. The swimming capability of fish estimated in the enclosed chamber is generally categorized into three modes, induced (U_ind_), critical (U_crit_), and sprint (U_sprint_) swimming speeds, which contribute to the design criteria of fishway hydraulics [17,19,20,21]. However, recent studies have challenged the evaluation of fish swimming ability in enclosed swim chambers, since this approach may underestimate the actual burst capacity, resulting in an overly conservative fishway flow velocity design that may impede efficient passage [4,22,23,24]. Some experiments have confirmed that fish naturally swim at faster burst speeds in open-channel flumes than in confined chambers [21,25,26]. However, most studies on open-channel swimming behavior were performed on *Cypriniformes* or *Salmoniformes* species distributed in Europe as well as North and South America, with individual fish tagged and exposed to a single flow velocity [4,22,25,26]. Little is known about the relationship between hydraulics and fish volitional swimming behavior in open-channel flumes with different flow regimes. Therefore, additional information on natural fish swimming behavior, preferably for untagged fish to allow for more realistic results, is needed to support river restoration efforts.

*Schizothorax prenanti,* Tchang, 1930 *(S. prenanti)* is a *Cyprinidae* species, locally known as ya-fish. *S*. *prenanti* is a potamodromous and economically valuable species that lives primarily at the interface between torrents and subcritical flows [27]. During the reproductive season (from March to July), sexually mature individuals migrate from the Minjiang and Dadu Rivers to upstream tributaries for spawning [19]. In recent years, the wild population of *S. prenanti* has declined, mainly due to the development of cascade hydropower projects in the lower reaches of the Jinsha River in China [28,29]. Thus, research on their behavioral responses to various turbulent flows is required to design effective fishways to protect fish resources [30]. Few studies have been performed to evaluate the effects of realistic flow fields on the migratory behavior of this species. Given the plethora of interacting factors, it is extremely difficult to monitor fish swimming behavior in detail and to measure fine hydraulic variables in natural conditions. In comparison, these variables can be controlled in laboratory studies, allowing for direct observations of fish swimming behavior and accurate measurements of the hydraulic variables [31].

This research effort focuses on the volitional swimming behavior of *S*. *prenanti* in an open-channel flume with alternative flow regimes. The primary objectives of this study were (i) to quantify the preferred ranges of the hydraulic variables of *S*. *prenanti* under natural habitat, (ii) to provide insight into fish swimming strategies when confronted with diverse locomotory challenges, and (iii) to evaluate the swimming ability of *S*. *prenanti* in an open-channel flume. This study has broad implications not only for the design and optimization of fishways for specific fish species but also for its description of a novel approach for studying other potamodromous migratory fish species.

## 2. Materials and Methods

### 2.1. Experimental Apparatus

**Figure 1:**
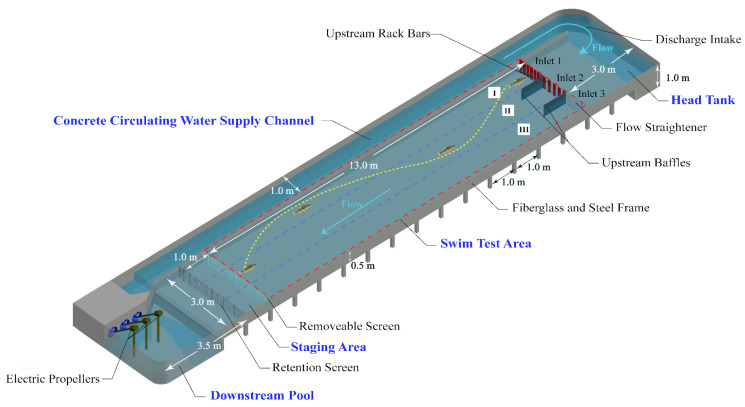
The geometric dimensions of the open-channel flume mainly including the head tank, swim test area, staging area, downstream pool, and concrete circulating water supply channel.

The fish volitional swimming behavior was investigated in an open-channel flume (20.5 m length × 4.0 m width × 1.0 m depth, zero slope) with a fiberglass and steel frame (Figure 1). The recirculatory flume had five functional parts: a flow-regulating head tank (3 m length × 3 m width × 1.0 m depth), a swim test area (13 m length × 3 m width × 0.5 m depth), a staging area (1 m length × 3 m width × 0.5 m depth), a trapezoidal downstream pool with approximate length and width of 3.5 m and 3.0 m, respectively, and a concrete circulating water supply channel on one side (18.5 m length × 1 m width × 1.0 m depth). Both the sides and the floor of the flume were smooth and straight in order to reduce flow friction and boundary layer effects.

A constant discharge (0.60 m^3^/s) was introduced from the water supply channel into the head tank by three electric propellers in the downstream pool (Figure 1). Specifically, the self-circulating power of the flume comes from the operation of the three downstream motors, and the rotation frequency of the motor is adjusted by inverter (adjustment range: 0–50 Hz). Three motors drive the rotation of three propellers consisting of three blades with a diameter of 0.36 m (see Figure S4a). A honeycomb flow straightener covering the entire width of the flume was placed upstream to align the flow direction to the swim test section (Figure 1). A series of rack bars (0.10 m length × 0.05 m width × 0.80 m height) were mounted to regulate the discharge that entered each inlet and to generate different velocity regimes downstream of the inlets. Specifically, seven, five, and zero rack bars were installed at inlets 1, 2, and 3, which formed flow regimes I, II, and III, respectively (Figure 1). In order to show the characteristics of flow velocity in regimes I, II, and III, we also defined these regimes as low-, moderate-, and high-flow regimes, respectively. Additionally, two baffles were installed to prevent the intermixing of different flow regimes downstream of the inlets (Figure 1). Water leaving the swim test area flowed into the staging area, which contained two screens for fish acclimatization (Figure 1), before returning to the downstream pool. Before the start of the experiment, water with a depth of 30 cm was stored in the flume. During the experiment, the water level varied within the range of 35–40 cm. An electromagnetic flowmeter and a water-level stylus were used to measure the flow discharge and water depth, respectively. The flow discharge and depth were kept constant by setting the same rotational speed of the electric propellers for all trials.

### 2.2. Hydraulic Characteristics

**Figure 2:**
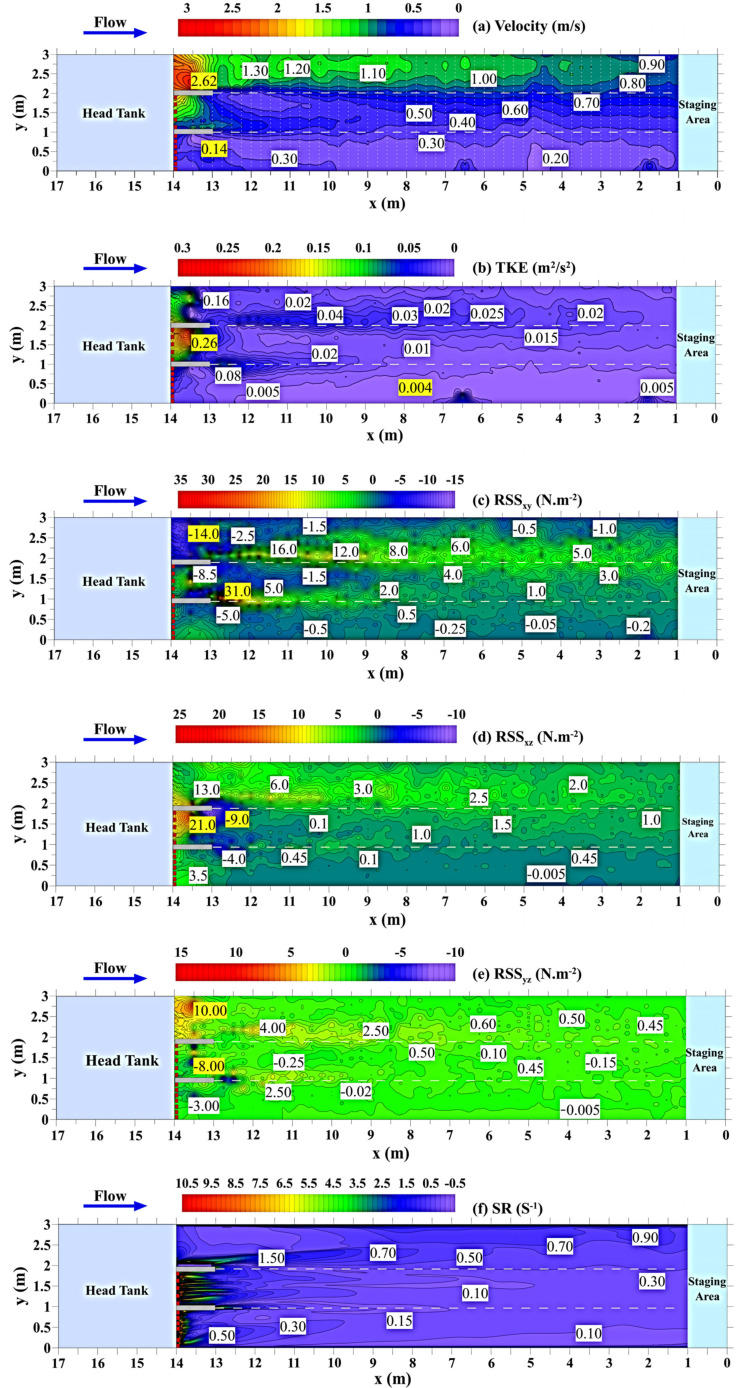
Contour plots of flume hydraulics and tolerances: (**a**) flow velocity (m/s), with the white dots indicating ADV measurement points, the white dashed lines indicating the flow regime boundaries, and the yellow labels in each contour plot indicating the minimum and maximum values of hydraulic factor, (**b**) TKE (m^2^/s^2^), (**c**) RSS_xy_ (N·m^−2^), (**d**) RSS_xz_ (N·m^−2^), (**e**) RSS_yz_ (N·m^−2^), and (**f**) SR (s^−1^).

Detailed instantaneous flow field components along the longitudinal (*u*), lateral (*v*), and vertical (*w*) directions in the swim test area (red dotted rectangle in Figure 1) were measured by a high-resolution acoustic doppler velocimeter (ADV) (Nortek As, Rud, Norway) at a frequency of 50 Hz for 60 s. Measurements were conducted prior to all fish trials to avoid the flow field being affected by fish swimming behavior. The velocity was measured at 10 cm above the bottom of the flume, since visual observation through the glass sidewall revealed that the fish basically swam in this horizontal plane. The longitudinal (*x*) and lateral (*y*) measurement distance intervals were 0.25 m and 0.1 m, respectively, and a total of 1421 measurement locations were recorded (white dots in Figure 2a). The raw ADV data were postprocessed using the Win-ADV program to control the quality of the timeseries data [32].

The focal flow velocity can be expressed as the sum of the time-averaged ($\bar{u}$, $\bar{v}$, $\bar{w}$) and fluctuating ($u^{\prime }$, $v^{\prime }$, $w^{\prime }$) components. Flow turbulence, including the TKE and RSS, impacts fish behavior and depends on vortex structures with various shapes and sizes [33]. The TKE reflects the kinetic energy of flow fluctuations and is defined as

(1) $$K=(\bar{u^{\prime 2}}+\bar{v^{\prime 2}}+\bar{w^{\prime 2}})/2.$$

RSS appears when two water masses or layers with different velocities are adjacent and can be partitioned into three planes: horizontal (*xy* plane), vertical (*xz* plane), and transversal (*yz* plane). The horizontal component of the RSS is defined as follows, and the vertical and transversal components of the RSS were calculated similarly:

(2) $$\tau_{uv}=-\rho \bar{u^{\prime }v^{\prime }}.$$

The computational fluid dynamics (CFD) software Flow-3D was used to simulate the fine flow field in the flume (Figures S1–S3) to determine an additional hydraulic variable, the strain rate (SR). The SR reflects the deformation degree of turbulent flow [34] and is defined as

(3) $${SR}_{ij}=(\frac{\partial u_{i}}{\partial x_{j}}+\frac{\partial u_{j}}{\partial x_{i}})\frac{1}{2}.$$

### 2.3. Fish Species Husbandry and Trials

Sexually mature [35] and cultured *S*. *prenanti* specimens (*n* = 60) were supplied by the Fisheries Institute of Sichuan Province on 10 October 2020 and held in three circular holding tanks (1500 L) sterilized with permanganate to minimize the risk of disease. The fish were fed with general fish food daily until 24 h before the experiment to reduce the impact of digestion on the test. Specifically, the protein level in the ration was 30%, and the amount provided daily was 2% of the fish biomass. During the experimental period, 10% of the water volume was replaced daily with aerated fresh water to maintain the water quality (DO > 6.5 mg/L, 7 < pH < 8), and medical-grade constant-temperature ice packs were used to maintain the water temperature (mean ± SD = 18.83 ± 0.24 °C).

All trials in the open-channel flume were conducted between 15 and 25 October 2020 during daylight hours (from 9:00 a.m. to 3:00 p.m.). Before each trial, a healthy individual fish was randomly seined from the holding tank into the staging area of the flume (Figure 1) to acclimate to the temperature and flow conditions for 0.5 h. Following this period, the removeable screen in the staging area (Figure 1) was raised to allow the fish to swim freely in the test area which included regimes I, II, and III at the same time (Figure 1), and their swimming behavior was continuously recorded using four video cameras (WIM SkyStar, 30 fps) installed 4.0 m above the centerline of the open-channel flume.

The induced, critical, and burst swimming speeds of this species with similar body lengths (0.29 ± 0.01 m) were tested by Fu et al. (2013) [36] in an enclosed swim chamber under a comparable water temperature (mean: 19.40 °C). In the swim test area, the flow velocity range (from minimum to maximum speed) in the whole area of regime I was marginally higher than the induced swimming speed (0.01–0.13 m/s) of *S*. *prenanti* [36]. In flow regime II, which had fewer rack bars than regime I, the flow velocities were similar to the critical swimming speed (0.65–1.09 m/s) [36]. In flow regime III, which had no rack bars, the flow velocity was within the burst swimming ability (0.85–1.53 m/s) range [36]. Before the formal experiment, a series of preliminary experiments were conducted to set suitable flow velocities range for testing fish volitional swimming behavior. In the preliminary experiments, the fish consistently exhibited negative rheotaxis behavior and employed prolonged or sprint swimming modes to negotiate various turbulent flows, which confirmed that the velocity settings and flume dimensions were appropriate for fish volitional swimming. The test ended when the fish passed through the entire test area or never left the staging area after 1 h (*n* = 8). The experimental data for each of the remaining test fish (*n* = 52) that successfully swam in the test area were analyzed. After each trial, the length (mean ± SD: body length (*BL*) = 26.9 ± 2.4 cm, fork length (*FL*) = 29.6 ± 2.5 cm, total length (*TL*) = 32.3 ± 2.7 cm) and weight (mean ± SD = 304.21 ± 77.15 g) of each fish were measured. The experiments were conducted under constant hydraulic conditions with 60 replicates (see Table S1). In order to avoid behavioral interference between different tests, each fish was tested only once.

### 2.4. Swimming Behavior

#### 2.4.1. Swimming Path

**Figure 3:**
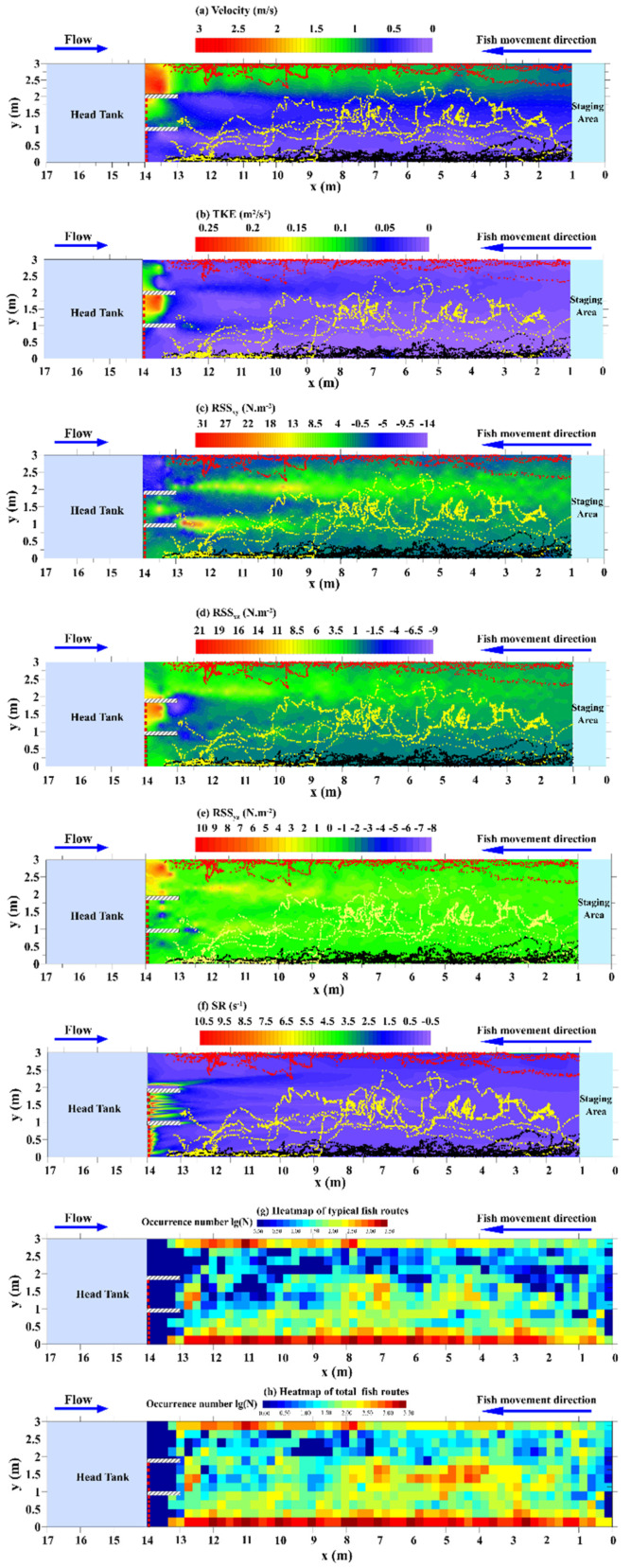
Typical fish swimming routes (**a**–**c**) against the contour plots of (**a**) flow velocity (m/s), (**b**) TKE (m^2^/s^2^), (**c**) RSS_xy_ (N·m^−2^), (**d**) RSS_xz_ (N·m^−2^), (**e**) RSS_yz_ (N·m^−2^), and (**f**) SR (s^−1^), as well as heatmaps of the fish occupancy number (lg(N)) in each cell: (**g**) typical fish swimming routes and (**h**) swimming routes of all fish.

Logger Pro 3.16 (Vernier, Beaverton, OR, USA) was used to determine the locations of the fish in two dimensions while they moved. The consecutive frames (30 fps) of the recorded video were analyzed, and the trajectories of individual fish were extracted by connecting their locations in chronological order. The typical fish swimming routes were classified as routes a, b, and c in Figure 3a–f, with each path representing the route of an individual fish. For typical route a (black dots), the fish moved slowly along the side in flow regime I the entire time; for route b (yellow dots), the fish randomly crossed into the low- or moderate-flow regime zones and always avoided the high-flow regime; for route c (red dots), the fish moved quickly along the side in flow regime III to reduce passage time (Figure 3a–f). Routes that were not characterized by the above three typical routes or where the same fish did more than one movement in the same trial were defined as other routes.

The fish ground speed ($V_{ground}$) between consecutive frames can be calculated as

*V_ground_* = *s*/Δ*t*, (4)

where s is the distance that the test fish moved relative to the geodetic coordinate system between consecutive frames, and Δ*t* (1/30 s) is the time taken to cover that distance. The swim test and staging areas (14 m length × 3 m width) were divided into 672 square cells (Figure 3g,h and Figure 6a) with dimensions of 0.25 m × 0.25 m, defined by the average fish ground speed in 1 s ($V_{ground}$ × 1 s). Heatmaps (Figure 3g,h) were used to visualize the fish occupancy count, normalized by a logarithmic (lg(N)) transformation, and the impact of the hydraulic variables on the fish transit time was analyzed in each cell.

#### 2.4.2. Swimming Strategy

According to the recorded videos, the fish swimming strategies were classified into three types: gaits 1, 2, and 3 (Figure 5a). Gait 1 represented rhythmic small tail-beat amplitude and fish body movement; gait 2 represented slight wiggling of the body and caudal fin for propulsion; gait 3 represented vigorous contraction of the fish body and tail muscles to achieve better thrust and acceleration than the first two gaits (Figure 5a). The heatmap in Figure 6a shows the maximum value (1, 2, or 3) of the gait employed by the individual fish that successfully navigated the entire flume length in each cell. For example, if different fish exhibited gaits 1, 2, and 3 in the same cell, we defined the value of that cell as the maximum value of 3 to characterize the swimming kinematics.

#### 2.4.3. Swimming Capability

The flow velocities that stimulated fish to swim upstream against the current were defined as “induced flow” (white dots in Figure 7a). Because each fish selected an appropriate flow velocity range for swimming, we defined the flow velocities of the swimming paths as “preferred flow” (pink dots in Figure 7a). In addition, the flow velocities at which fish terminated movement were defined as “schooling flow” (black dots in Figure 7a).

The fish swimming speed [4] (relative to flowing water) is defined as

(5) $$V_{fish}=V_{ground}+V_{water},$$

where *V_water_* is the average water velocity against which the fish swam. For the low- and moderate-flow regimes, the average and 95% confidence intervals of all fish swimming speeds in each 0.1 m interval along the longitudinal (*x*) length of the swim test area are shown in Figure 8a,b, respectively. For the high-flow regime, the maximum burst swimming speed observed in each 0.1 m interval was extracted to show the anaerobic swimming ability in Figure 8c. The average fish swimming speed for all fish in the different flow regimes is shown in Figure 8d (black bars), and the fish passage time shown in Figure 8d (red bars) indicates the elapsed time (s) taken to traverse the entire swim test area.

### 2.5. Data Analysis

The Spearman rank coefficient was used to evaluate the correlations between the fish transit time in each cell (cell transit time) and the hydraulic variables (V, TKE, RSS) obtained from the ADV measurements and the SR obtained from the CFD simulation results. The Kruskal–Wallis analysis of variance (ANOVA) method was employed to assess the impact of the different flow velocity regimes (low, moderate, and high) on the fish ground speed and swimming speed. All statistical analyses were performed using IBM SPSS Statistics 22 software (IBM, New York, NY, USA), and all tests were two-sided with a significance level of 0.05.

## 3. Results

### 3.1. Flume Hydraulics and Tolerances

The flow velocity in the flume varied substantially among the different flow regimes, with average entrance velocities of 0.34, 0.90, and 1.68 m/s at inlets 1, 2, and 3, respectively (Figure 2a). In flow regime I, which had the highest density of rack bars, the flow velocity distribution was relatively uniform, and the magnitude of the velocity ranged from 0.14 to 0.38 m/s, which was lower than in the other flow regimes. The minimum flow velocity (approximately 0.14 m/s) was observed at the recirculation region near inlet 1. In flow regime II, which had fewer rack bars, the flow field became nonuniform from left to right and exhibited some “band” patterns. The longitudinal flow velocities increased from 0.33 to 0.78 m/s with increasing distance from the inlet (Figure 2a). For intake 3, which had no rack bars (Figure 1), the flow velocity (0.86–2.62 m/s) increased in the upper test area and then gradually decreased in the downstream direction.

As illustrated in Figure 2b, similar to the flow velocity, the spatial distribution and magnitude of the TKE differed in the three flow regimes. Small TKE values on the order of 0.005 m^2^/s^2^ were observed in flow regime I, which had quasi-uniform and small velocity distributions. However, in regime II, the flow was nonuniform, resulting in increasing TKE values towards the inlet (0.01–0.25 m^2^/s^2^). The mean TKE value was approximately 0.03 m^2^/s^2^ in flow regime III increasing along the test area from <0.02 m^2^/s^2^ at the downstream end to > 0.16 m^2^/s^2^ at the upper end, and the oval-shaped regions of higher turbulence tended to move further downstream.

The RSS values in the three planes (RSS_xy_, RSS_xz_, RSS_yz_) shown in Figure 2c–e indicate that the RSS value generally increased with increasing flow discharge at the three inlets. The minimum and maximum values of RSS_xy_ in the flume were −14 and 31 N·m^−2^, respectively, which were observed near the intake areas (Figure 2c). Negative RSS_xy_ values were primarily distributed near both sidewalls. The minimum (−9 N·m^−2^) and maximum (21 N·m^−2^) RSS_xz_ values were observed near intake 2, and RSS_xz_ ranged from approximately −0.005 to 3.5 N·m^−2^ in the test area (Figure 2d). Compared to RSS_xy_ and RSS_xz_, RSS_yz_ was generally lower in the flume and ranged from −8 to 10 N·m^−2^ (Figure 2e). The SR values in the main research area ranged from 0.1 to 1.5 s^−1^ (Figure 2f).

### 3.2. Spatial and Temporal Occupancy of the Hydraulics

The volitional swimming behavior of 60 fish samples was investigated in the open-channel flume; 52 fish successfully moved into the test area, while eight never left the staging area. For the routes (*n* = 52) recorded in the swim test area, the proportions of routes a, b, and c were 32.69%, 28.85%, and 13.46%, respectively, with other routes accounting for 25.00%. The fish swimming trajectories were overlaid on the hydraulic profiles to further analyze the preferred ranges of the hydraulic variables (Figure 3a–f). Heatmaps of typical (*n* = 39) and all (*n* = 52) fish swimming routes are presented in Figure 3g,h, respectively.

**Figure 4:**
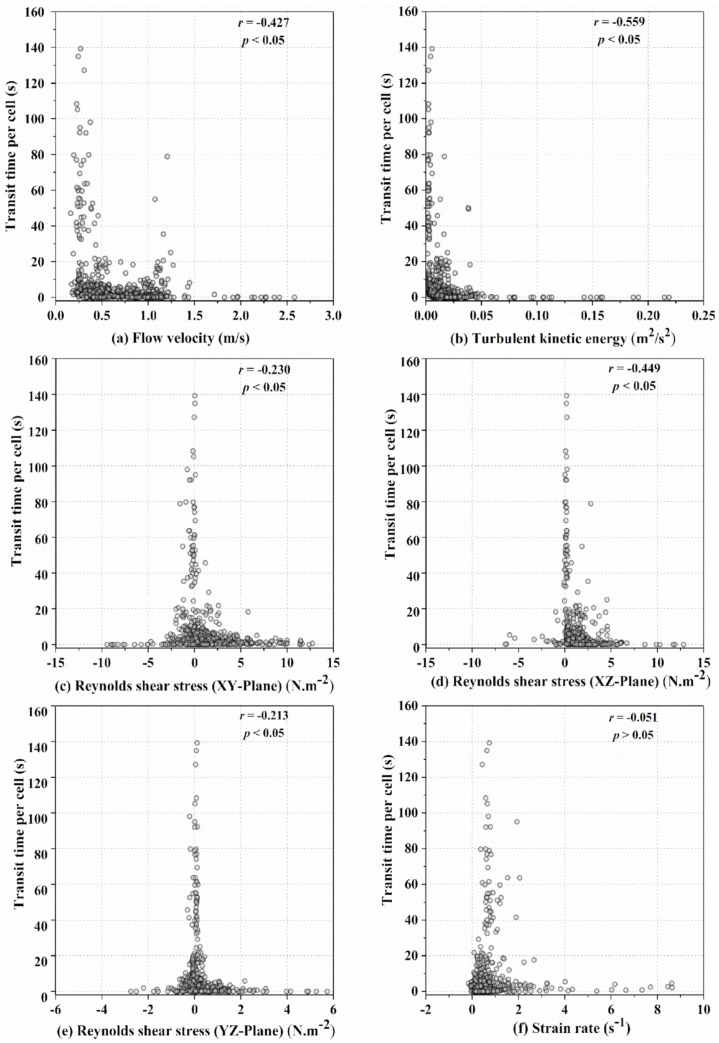
Distribution of fish transit time in cells versus (**a**) flow velocity (m/s), (**b**) TKE (m^2^/s^2^), (**c**) RSS_xy_ (N·m^−2^), (**d**) RSS_xz_ (N·m^−2^), (**e**) RSS_yz_ (N·m^−2^), and (**f**) SR (s^−1^).

The relationship between the average values of the hydraulic characteristics and the respective fish transit times in each cell is shown in Figure 4. Most of the fish preferred to ascend in areas with low flow velocities, ranging from approximately 0.25 to 0.5 m/s (Figure 4a), resulting in a statistically significant negative correlation between the flow velocity and fish transit time (Spearman rank correlation: *r* = −0.427, *p* < 0.05). The TKE values for the selected swimming paths were also low (< 0.05 m^2^/s^2^). Additionally, fish tended to avoid regions with high TKE values, ranging from 0.05 to 0.25 m^2^/s^2^ (Figure 4b). There was a statistically significant negative correlation between the magnitude of the TKE and the fish transit time (Spearman rank correlation: *r* = −0.559, *p* < 0.05), with an absolute *r* value slightly higher than that for flow velocity.

The preferred range of RSS_xy_ was from −5 to 5 N·m^−2^, as shown in Figure 4c, and a statistically significant negative correlation between RSS_xy_ and transit time was found (Spearman rank correlation: *r* = −0.230, *p* < 0.05). Fish spent more time in cells with lower RSS_xz_ values (0–5 N·m^−2^). As a result, there was a statistically significant negative correlation (Figure 4d) between RSS_xz_ and the fish transit time (Spearman rank correlation: *r* = −0.449, *p* < 0.05). The preferred range of RSS_yz_ was approximately −1 to 2 N·m^−2^ (Figure 4e), and a statistically significant negative correlation was found between RSS_yz_ and transit time (Spearman rank correlation: *r* = −0.213, *p* < 0.05). Moreover, fish primarily used cells with low SR values (0–2 s^−1^), but no statistically significant correlation (Figure 4f) was found between the SR and transit time (Spearman rank correlation: *r* = −0.051, *p* > 0.05).

### 3.3. Varied Swimming Strategy

**Figure 5:**
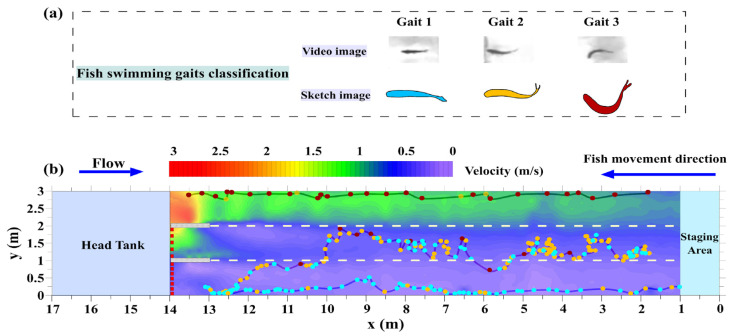
(**a**): Classification of fish swimming gaits and (**b**) the typical swimming routes with different gaits against the flow velocity (m/s). The different colored dots indicate the different gaits classified in (**a**).

Three representative swimming routes of different individual fish were selected to describe the characteristics of the fish swimming strategy in the different flow regimes; the time interval between two consecutive points was 1 s (Figure 5b). In regions with relatively slow currents, fish that moved slowly along the wall with gaits 1 and 2 were observed to stagger forward. In the moderate-flow regime, fish primarily exhibited searching behavior in gaits 1 and 2, minimizing their exposure to high-velocity currents. However, in the high-velocity regime, the fish changed their swimming strategies, minimizing the time spent searching and increasing their swimming speed to expedite passage. As a result, in this region, gaits 2 and 3 were used by fish for burst-coast behavior.

**Figure 6:**
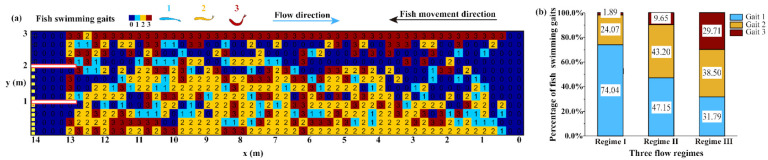
Quantification of fish swimming gaits in the swim test area: (**a**) heatmap of the maximum gait value in each cell employed by the swimming fish; (**b**) percentage of the different fish swimming gaits in the three flow regimes.

As shown in Figure 6a, most of the fish that successfully crossed the entire length of the flume swam in the low- and moderate-flow velocity regions with maximum gait values of 1 and 2. In the higher-flow regime, fish quickly ascended along the wall with gait 3 to save time. The total count of the different gaits employed by the fish is quantified in Figure 6b. In flow regime I, gait 1 (74.04%) was used more frequently than gait 2 (24.07%) and gait 3 (1.89%). In flow regime II, the percentages of gaits 2 and 3 increased to 43.20% and 9.65%, respectively. In flow regime III, gait 3 was more prominent, with a remarkable increase to 29.71%, due to the fish navigating the higher flow velocities.

### 3.4. Volitional Swimming Ability

**Figure 7:**
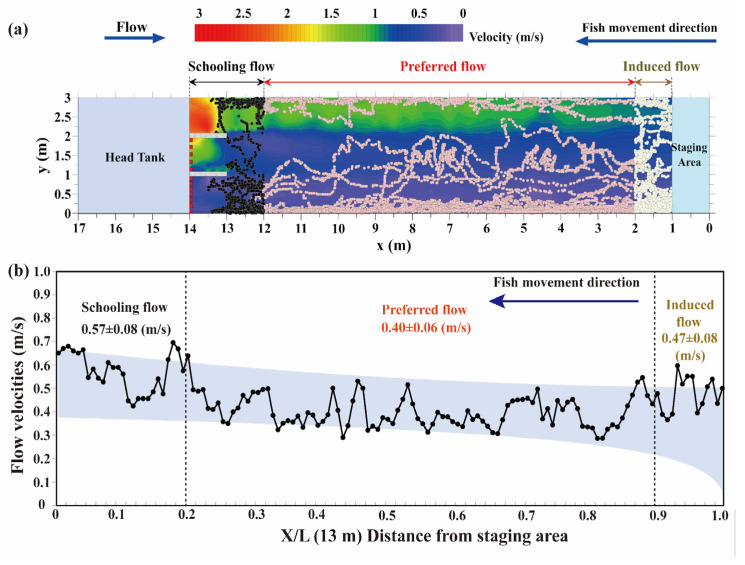
(**a**): Classification of the flow velocity as induced, preferred, and schooling flow based on the different fish swimming schemes; (**b**) 95% confidence intervals of the flow velocity range and fish movement distance.

**Figure 8:**
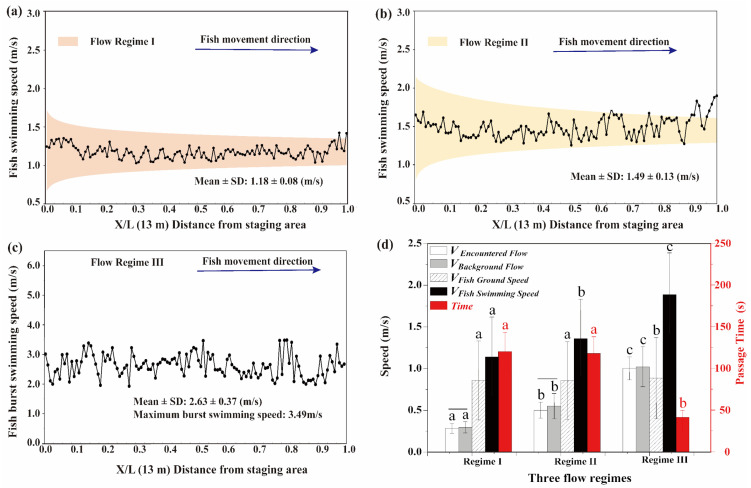
The relationship between the average and 95% confidence intervals of fish swimming speed (m/s) with movement distance in (**a**) regime I and (**b**) regime II, and the maximum burst swimming speed in (**c**) regime III; (**d**) encountered flow (m/s), background flow (m/s), fish ground speed (m/s), fish swimming speed (m/s), and passage time in the different flow regimes, with the difference in the same variable indicated by different letters (a, b, and c), and the difference between the encountered flow and background flow indicated by the black solid line.

The estimated flow velocities at different stages during the fish upstream movement for yielding induced, preferred, and schooling flows (Figure 7a) were 0.47 ± 0.08, 0.40 ± 0.06 m/s, and 0.57 ± 0.08 m/s, respectively (Figure 7b). Moreover, the fish volitional swimming abilities differed in the various flow regimes (Figure 8a–c). Specifically, the fish swimming speeds in the low- and moderate-flow regimes were 1.18 ± 0.08 (Figure 8a) and 1.49 ± 0.13 m/s (Figure 8b), respectively. In the high-flow regime, the average burst swimming speed was 2.63 ± 0.37 m/s, with a maximum value of 3.49 m/s (Figure 8c).

There was a statistically significant difference (*p* < 0.05) between the flow velocities encountered by fish in regime III (V _Encountered Flow_ = 1.01 ± 0.14 m/s) and in regime I (V _Encountered Flow_ = 0.28 ± 0.06 m/s) and regime II (V _Encountered Flow_ = 0.50 ± 0.10 m/s) (Figure 8d, white bars). The flow velocity values that the different regimes had originally were defined as the “background flow” to distinguish it from the encountered flow. Furthermore, the mean background flow velocities (V_Background Flow_) in regimes I, II, and III were 0.29, 0.55, and 1.02 m/s, respectively (Figure 8d, gray bars). There was a statistically significant difference (*p* < 0.05) between the encountered and background flow velocities in regimes I and II (indicated by the black solid lines in Figure 8d). Additionally, there was a statistically significant (*p* < 0.05) difference between the fish ground speed in flow regime III (0.89 ± 0.49 m/s) compared to either regime I (0.85 ± 0.46 m/s) or regime II (0.86 ± 0.44 m/s) (Figure 8d, shaded bars). In addition, fish exhibited a faster swimming speed in regime III than in the other flow regimes (Figure 8d, black bars), resulting in a significantly (*p* < 0.05) shorter passage time in this regime (41.49 ± 8.19 s) than in regime I (120.22 ± 23.19 s) or regime II (117.89 ± 19.95 s) (Figure 8d, red bars).

## 4. Discussion

### 4.1. Effects of Hydrodynamic Characteristics on Fish Swimming Path Selection

Fish spatial and temporal occupancies, as well as the behavioral responses to varied flow conditions in an open-channel flume, were estimated to investigate the effect of flow turbulence characteristics on fish swimming behavior. Fish spent more transit time in regions with lower flow velocities (0.25–0.50 m/s) and avoided areas with higher flow velocities ranging from 1.5 to 2.5 m/s. These lower flow conditions were mainly observed near both sides and in flow regimes I and II, since the fish appeared to minimize their exposure to high-speed currents to avoid destabilizing behavior [16]. Previous studies have revealed that fish can use appropriate vortices generated by moderate turbulent flows to propel their forward movement or station-holding abilities, whereas higher turbulent flows may cause swimming instabilities and increase energy expenditure [8,10,28,37]. The preferred flow velocity range, determined by the volitional swimming performance, can be used to evaluate the habitat suitability of *S*. *prenanti* and estimate the fish resources under specific hydrological conditions [38]. Furthermore, this range can be used as a reference for designing roughness elements or slot positions in fishways to create appropriate hydraulic characteristics that allow fish to pass [28].

Similar to the flow velocity trends, *S*. *prenanti* was observed to mainly occupy areas with low TKE values (<0.05 m^2^/s^2^) and avoid regions with high TKE values ranging from 0.05 to 0.25 m^2^/s^2^. This was consistent with the observation that TKE values of 0.05 m^2^/s^2^ did not stress rainbow trout [13] and with the results of Silva et al. (2020) [8] that TKE values less than 0.03 m^2^/s^2^ in main river flows were suitable for Atlantic salmon to perform steady swimming, in contrast to high TKE values (0.03–0.24 m^2^/s^2^), which increased locomotory costs [39] or stress (0.22 m^2^/s^2^) [13]. Although fish encounter various turbulent conditions in natural rivers, only certain properties (e.g., scale, magnitude, orientation, intensity, and periodicity) affect fish behavior, aggregation, and migration [12]. The fish behavioral response to the turbulence threshold conditions should be investigated further by combining laboratory and field studies on different fish species to design and refine river restoration schemes.

The value of the Reynolds shear stress, which indicated the effect of turbulent momentum exchange on fish behavior, differed for various shear stress components (*xy*, *xz*, and *yz* planes) in this study. Specifically, the interaction between the fish transit time and the vertical Reynolds shear stress (*xz* plane) was the strongest among the flow velocity and three shear stress components, implying that this variable is important in fish–turbulence interaction studies. For the SR, although the result was not statistically significant, the fish spent more time in regions with lower SR values ranging from 0 to 2 s^−1^, which was consistent with Tan et al. (2019) [40], since a higher turbulent dissipation rate and SR may cause fish to lose balance and even overturn [33].

### 4.2. Fish Swimming Strategy Response to Varied Flow Regimes

In this study, *S*. *prenanti* primarily employed gaits 1 and 2 for maneuvering and stabilization in the low- and moderate-flow regimes and then transitioned to gait 3 in higher-flow regions to navigate challenging flows. The swimming paths in gaits 1 and 2 tended to be more meandering, while there were more linear trajectories in gait 3. Furthermore, in the low- and moderate-flow regimes, *S*. *prenanti* actively selected even lower speed current pathways, resulting in a statistically significant difference between the encountered and background flow velocities. In higher-flow regions, the fish did not search for lower-speed pathways, instead encountering the average or even higher flow speeds. These results are consistent with previous field work conducted by Standen et al. (2004) [16]. Furthermore, the fish swimming speed in the high-flow regime was significantly higher than that in the low- and moderate-flow regimes. In regions with higher flow velocities, fish may change their swimming strategy from energy conservation to time conservation by minimizing the amount of time spent searching for lower velocity areas and increasing their swimming speed to more quickly complete their passage. Notably, fish may trade heavy exercise intensity for minimizing exercise duration as an evolutionary strategy to conserve anaerobic fuel supply and reduce postexercise oxygen debt when faced with high flow velocities [16]. In the future, it is necessary to study the fatigue time and physiological metabolism of different swimming patterns, such as burst–coast or burst–prolonged swimming, as well as hydraulic thresholds that trigger switching to different modes, to better understand the behavioral responses of fish to complex flow fields.

### 4.3. Implications of Volitional Swimming Capability on Fishways

The volitional swimming capability of *S*. *prenanti* in an open-channel flume was quantified, and the average and maximum burst swimming speeds in the high-flow regime were 2.63 ± 0.37 and 3.49 m/s, respectively. These values were 2.21- and 2.28-fold more than the average (1.19 m/s) and maximum (1.53 m/s) burst speeds, respectively, estimated in an enclosed swim chamber for individuals of this species [36]. The greater-than-forced sprinting speed is important for fishway design since fish often use this speed to navigate short high-velocity segments of fishways, such as flows over weirs, between rocks, or through vertical slots [24], whereas flow velocities exceeding 3.49 m/s in fishways may be identified as a potential barrier for this species. Moreover, the swimming speeds measured in the low- (1.18 ± 0.08 m/s) and moderate-flow (1.49 ± 0.13 m/s) regimes were higher than the critical swimming speed (mean: 0.87 m/s) tested by Fu et al. (2013) [36]. Sanz-Ronda et al. (2015) [4] and Kern et al. (2018) [23] also found that, in the burst range, fish performed better in open channels or large recirculating flumes than in swim chambers, which may be more appropriate for prolonged fish speeds as indicated by Katopodis and Gervais 2016 [21].

The natural swimming behavior of fish may be limited by the relatively small, enclosed swim chamber, causing earlier fatigue [41], which may explain some of the differences in fish speed estimates between open channels and enclosed swim chambers, particularly in the burst swimming range. In this study, *S*. *prenanti* employed gaits 2 and 3 for sprinting upstream and gliding in higher-flow regimes, supporting the conclusion that the gait transition from steady swimming mode to burst–coast swimming mode for processing metabolic waste was regarded as an energetic advantage [42]. However, Sanz-Ronda et al. [4] observed that the swimming endurance and speed of barbel (*Luciobarbus bocagei*) and nase (*Pseudochondrostoma duriense*) with consistent steady swimming behavior in open-channel flumes greatly exceeded the result obtained in enclosed chambers. This could be due to fish selecting different ground speeds to navigate variable flow conditions [43,44]. In addition, the difference in hydrodynamic conditions between enclosed swim chambers and open-channel flumes may have a significant impact on fish swimming performance [33]. In an enclosed flume, the greater flow variability in the cross-section may result in a higher or lower estimate of the fish swimming ability, depending on whether the fish can identify low-flow-velocity regions [23]. Given the conservative estimates of fish swimming ability obtained in enclosed swim chambers, understanding the causes of these differences may help researchers to appropriately extrapolate data for fish swimming ability estimated from laboratory studies to natural situations.

Generally, higher flow velocities are needed for the positive rheotactic behavior of fish to find and enter fishways [45,46]. In this paper, we quantified the flow velocities in the starting area with an average induced flow velocity of approximately 0.47 m/s, which was between the induction (0.1 m/s) and critical (0.87 m/s) swimming speeds estimated from the enclosed chamber [39]. This was consistent with previous work in which Pavlov (1989) [47] and Cai et al. (2020) [48] recommended a ratio of the entrance velocity of the fishway to the fish critical swimming speed between 0.6 and 0.8. Within fishway facilities where fish need to continuously swim, endurance and maximum traversable swimming speed should be considered [17]. We quantified the flow velocities on the basis of individual fish swimming pathways and found that the average flow magnitude was approximately 0.4 m/s. In addition, we estimated the flow velocities where fish terminated movement (mean: 0.57 m/s) and defined it as the schooling velocity. These results were consistent with previous studies [24] that indicated that appropriate fishway velocities in passageways or resting pools should be between the induction and critical swimming speeds to allow fish to recover from sprint behavior.

The findings of this study, including the preferred hydraulic characteristics, swimming strategies, and swimming capabilities of fish, provide a novel approach to fill the knowledge gap in volitional swimming behavior studies on *Schizothorax* fish species under more realistic flow conditions. However, the results of this research may be limited by the fish samples without tagged and high variability in velocity along the fish swimming trajectory caused by the experimental apparatus; thus, some additional swimming performance data, including fatigue time or metabolism transition which affect fishway efficiency was not assessed here. Furthermore, the effect of using cultured fish with less migratory motivation on volitional swimming behavior is uncertain. Future studies are needed to understand the natural behavior by testing several wild species with strong migratory motivation in controlled laboratory experiments and more realistic water flow conditions.

## 5. Conclusions

The volitional swimming behavior associated with path selection, behavior strategies, and swimming speed of *S*. *prenanti* was studied in an open-channel flume with more realistic flow patterns than an enclosed chamber. The hydrodynamic temporal and spatial occupancies varied with the flow velocity, TKE, RSS, and SR. In this study, *S*. *prenanti* spent more transit time in regions with relatively low flow velocities (0.25–0.50 m/s) and turbulence (<0.05 m^2^/s^2^), while they avoided zones with relatively high flow velocities (1.5–2.5 m/s) and turbulence (0.05–0.25 m^2^/s^2^). The characteristics of the turbulence had statistically significant effects on the fish swimming gait response. Specifically, *S*. *prenanti* primarily employed steady swimming behavior in low- and moderate-flow regimes to search for flow velocities lower than the average current as an energy conservation strategy. However, in the higher-flow regime, *S*. *prenanti* mostly exhibited unsteady sprint behavior to minimize exercise duration. Moreover, we quantified the volitional swimming speed of *S*. *prenanti* and found that the average and maximum burst swimming speeds were 2.63 ± 0.37 and 3.49 m/s, respectively. These values were 2.21- and 2.28-fold higher than the average (1.19 m/s) and maximum (1.53 m/s) burst speeds estimated from an enclosed swim chamber. This research has important implications for improving the understanding of fish behavioral responses to various turbulent flows and providing useful recommendations for the design and optimization of fishways.

## Data Availability

The data that support the findings of this study are available from the corresponding author upon reasonable request.

## Supplementary Materials

The following supporting information can be downloaded at: https://www.mdpi.com/article/10.3390/ani12060752/s1, Figure S1: Contour plot of flow velocity (m/s); Figure S2: Three-dimensional velocity vector; Figure S3: Comparison of the measurement and simulation flow velocity (m/s) results. The dashed line represents 20% error; Figure S4: On-site photos of experiment; Table S1: Swimming speed (m/s ± SD) and ascent time (s) for each fish (*n* = 60) under different trajectories.
